# Record Review to Explore the Adequacy of Post-Operative Vital Signs Monitoring Using a Local Modified Early Warning Score (Mews) Chart to Evaluate Outcomes

**DOI:** 10.1371/journal.pone.0087320

**Published:** 2014-01-31

**Authors:** Una Kyriacos, Jennifer Jelsma, Sue Jordan

**Affiliations:** 1 Department of Health & Rehabilitation Sciences, Faculty of Health Sciences, University of Cape Town, Cape Town, South Africa; 2 School of Human and Health Sciences, Swansea University, Swansea, Wales, United Kingdom; D’or Institute of Research and Education, Brazil

## Abstract

**Objectives:**

1) To explore the adequacy of: vital signs’ recordings (respiratory and heart rate, oxygen saturation, systolic blood pressure (BP), temperature, level of consciousness and urine output) in the first 8 post-operative hours; responses to clinical deterioration. 2) To identify factors associated with death on the ward between transfer from the theatre recovery suite and the seventh day after operation.

**Design:**

Retrospective review of records of 11 patients who died plus four controls for each case.

**Participants:**

We reviewed clinical records of 55 patients who met inclusion criteria (general anaesthetic, age >13, complete records) from six surgical wards in a teaching hospital between 1 May and 31 July 2009.

**Methods:**

In the absence of guidelines for routine post-operative vital signs’ monitoring, nurses’ standard practice graphical plots of recordings were recoded into MEWS formats (0 = normal, 1–3 upper or lower limit) and their responses to clinical deterioration were interpreted using MEWS reporting algorithms.

**Results:**

No patients’ records contained recordings for all seven parameters displayed on the MEWS. There was no evidence of response to: 22/36 (61.1%) abnormal vital signs for patients who died that would have triggered an escalated MEWS reporting algorithm; 81/87 (93.1%) for controls. Death was associated with age, ≥61 years (OR 14.2, 3.0–68.0); ≥2 pre-existing co-morbidities (OR 75.3, 3.7–1527.4); high/low systolic BP on admission (OR 7.2, 1.5–34.2); tachycardia (≥111–129 bpm) (OR 6.6, 1.4–30.0) and low systolic BP (≤81–100 mmHg), as defined by the MEWS (OR 8.0, 1.9–33.1).

**Conclusions:**

Guidelines for post-operative vital signs’ monitoring and reporting need to be established. The MEWS provides a useful scoring system for interpreting clinical deterioration and guiding intervention. Exploration of the ability of the Cape Town MEWS chart plus reporting algorithm to expedite recognition of signs of clinical and physiological deterioration and securing more skilled assistance is essential.

## Introduction

### Background

Adverse events (AEs) affect nearly one in seven hospital in-patients in the USA and cause the death of more people than breast cancer or AIDS [1]. The world’s largest provider of health care (Medicare) routinely reviews case-notes [2] to improve quality of care. Since the publication of the Harvard Medical Practice Study [3] of New York hospitals, the Colorado-Utah Study [4] and the Quality in Australian Health Care Study [5] record review has become the mainstay of quality assurance measures.

This paper considers AEs as failure to rescue acutely ill patients from physiological deterioration, that is: non-recognition of early signs of clinical deterioration, misinterpretation of clinical data and delayed response in summoning more skilled assistance or in attending to a call for assistance [6]. Post-operative patients require frequent, skillful monitoring of vital signs on general wards to avoid AEs. Although 70–80% of AEs in complex health care systems may be due to human error, organizational systems themselves contribute to the problem [5], [7] such as inadequate clinical guidelines, monitoring charts and rapid response systems. Unanticipated ICU admission and in-hospital death [8] have medico-legal consequences if found to be preventable.

The incidence of AEs and negligence of staff caring for hospitalized patients is receiving serious attention at national level in developed health care systems [9]–[11]. In the UK, older and more acutely ill patients are being cared for on general wards by fewer qualified nurses, who are not paid for study leave to attend post-registration education, and by more inexperienced, temporary nurses [12]. The association between vital sign parameters (fast pulse rate and low systolic BP) and mortality [13]–[15] challenges traditional assumptions that mortality outcomes and determinants of survival fall solely within the domain of medical care, and provides further evidence that these outcomes are ‘nursing sensitive’ [13]. Nurses’ concerns about caring for critically ill patients on general wards are that patients are having increasingly more complex surgery, increasing their dependence and morbidity, which, in the face of understaffing, results in increased workload and suboptimal quality of care, leaving less time to apply learning in practice [16], [17].

Although the primary function of bedside observations charts is to make clinicians aware of patients’ deterioration, performance of these charts is under-reported [18]. A variety of vital signs monitoring tools that incorporate early warning scoring (EWS) systems designed to track signs of deterioration and trigger a rapid response by more skilled clinicians to improve patient safety have been introduced in wards across the UK [19] and Australasia [10], [20]. The performance of aggregate weighted [21] and single parameter [8] ‘track and trigger’ EWS systems have been evaluated in observational studies. Following validation work, UK authorities advocate implementation of a standard national EWS (NEWS) system [22]–[24]. It is the nurses’ professional responsibility to understand the significance of patient observations [25] and patient survival often depends on the decisions of nurses to call for assistance.

EWS observations charts usually incorporate five to six physiological parameters each having a standardized range of cut points (for example heart rate 101–110 bpm) with corresponding colour-banded [26] weighted trigger points (0, upper and lower 1 to 3) [27]. The UK NEWS system [24] incorporates six parameters (respiratory rate, oxygen saturation, temperature, systolic blood pressure, pulse rate and level of consciousness). The weighted trigger points guide interventions for disturbed physiological values [28]–[30] for single parameters [31] and for aggregated MEWS systems [32].

**Figure 1:**
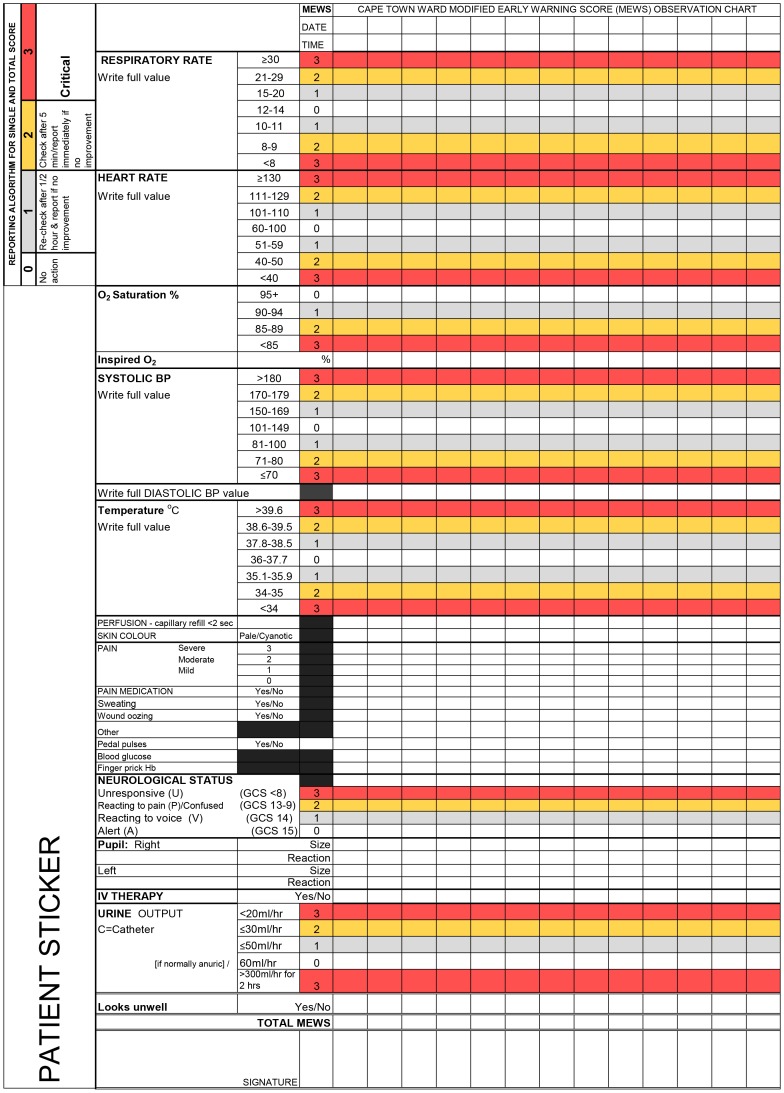
The Cape Town MEWS.

The Cape Town MEWS (Figure 1), a multidisciplinary consensus derived chart for general hospital wards, led by the first author, was designed over four months (September-December 2009) in response to concerns about the lack of clinical guidelines and reporting algorithms for clinical deterioration on standard observation charts. At the time of the study in 2009 no public hospital in the Western Cape Province in South Africa had used EWS systems on general wards. At the research setting the ‘cardiac arrest team’ comprised individual ward response teams. Clinical guidelines for activating the ward response teams were not located. The individual ward response system, rather than centralized critical care outreach or acute care teams, risked lack of consistency in the recognition of and response to clinical deterioration. Briefly, development of the local MEWS comprised two face-to-face consensus conferences (employing the nominal group technique) and three Delphi rounds (by electronic mail) with 8 to 11 experts (specialist anaesthesiologist, neurosurgeon, emergency medicine physician, critical care nurses and senior surgical nurses). The consensus derived local MEWS incorporated seven physiological parameters, each with colour-banded cut points (thresholds) and weighted trigger points (0 = normal, upper and lower 1–3 limits) and a response algorithm [33]. The local MEWS chart, unusually, also incorporated clinical signs of deterioration (for example pallor, sweating, looking unwell).

Standard ward bedside observations charts used in South African public hospitals during the study period required 4-hourly graphic plotting of temperature, pulse rate and blood pressure (standard parameters) but did not incorporate indicators of abnormality or a reporting algorithm. Respiratory rate was to be recorded on the admission chart. Some post-operative observations charts included the Glasgow Coma Scale for monitoring level of consciousness. Post-operative monitoring guidelines on the number of parameters or frequency of monitoring were not located and there were no clinical guidelines for the minimum standard of recording vital signs. Published evidence of the performance of South African standard ward observations charts in facilitating the detection of early signs of deterioration in a patient was not located.

Following development of the Cape Town MEWS, further research was needed to explore the ability of the chart to facilitate identification of clinical deterioration. It is the purpose of this paper to describe retrospective transfer of post-operative vital sign recordings from standard observation charts to the Cape Town MEWS for analysis and interpretation of the adequacy of immediate post-operative vital signs recordings in one public hospital in South Africa.

## Methods

### Ethical Considerations

The study was approved by the University of Cape Town, Faculty of Health Sciences Human Research Ethics Committee (REC REF 192/2009) and the research settings’ hospital management and clinical structures (withheld for confidential reasons but available upon request). The confidential nature of patient information, protection of anonymity and consent is paramount in record review. In this study institutional consent was obtained for record review. The research ethics committee approved the use of non-anonymous records as under South African legislation (Section 16 (2)) [34] health care providers may examine a service user’s health records for the purposes of research without authorization if the research will not identify the user. The research ethics committee and hospital management structures waived the need for consent from patients for the use of their records. Although reporting was anonymous, patients’ records were not, so all researchers signed a confidentiality clause.

Retrospective Record review is one of the main research methods for establishing the extent of adverse events (AEs) [35] and is not disruptive to delivery of health services [2].

### Design

Retrospective record review (1 May to 31 July 2009) of 11 case-notes of patients who had died and four controls for each case. The Strobe checklist (Table S1) was used to guide reporting of the study.

### Setting

Records were reviewed from 6 adult surgical wards in an 867-bed academic public hospital in Cape Town. During the study period there were 25,546 non-obstetric admissions and 1,502 deaths (5.9%). No early warning scoring system was in place on general wards. Patients in surgical wards having had general anaesthesia were selected as needing frequent vital signs monitoring.

### Participants

**Figure 2:**
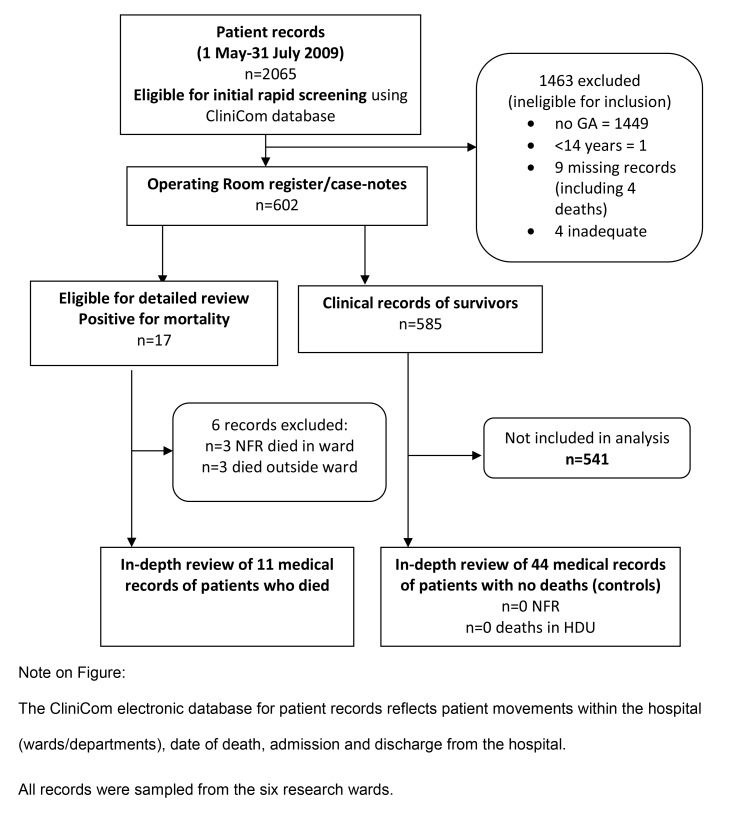
Record review flow chart.

Records of all patients >13 years of age [36] who had a general anaesthetic and were admitted to six purposively sampled adult wards for general, vascular and orthopaedic surgery between 1 May and 31 July 2009 were eligible for inclusion (Figure 2: Flow chart). Records of all those who suffered an unexpected death during the study period were selected. We included deaths without a pre-existing not-for resuscitation (NFR) order ([37], p. 2092). We included deaths occurring on the ward at any time up to 7 days after operation. We excluded deaths occurring outside the ward area such as High Dependency (HDU) and Intensive Care Units (ICU) where specialist nurses use continuous electronic monitoring systems and different observation charts.

Incomplete or unavailable records were excluded. Incomplete records were defined as not including either observations charts or patient progress notes. If either were absent, the case was excluded. Of the patients undergoing a GA who met all other inclusion criteria we excluded patients with a ‘not for resuscitation’ order, indicating that death was not unexpected, and who died outside the ward, in HDU as the study excluded patients not monitored on the ward. For the remaining patients, 4 controls were selected. These were the next four records on the hospital database where the patients had survived 7 days, had no ‘not for resuscitation’ orders and remained on the ward until discharge.

### Construction of Record Review Form

A local Cape Town consensus derived experimental MEWS incorporating a reporting algorithm [33] was used to recode patients’ vital signs from existing observations charts into a MEWS format for the purpose of interpreting severity of illness and appropriate responses. A record review form with explicit criteria was designed on a password-protected Excel© spreadsheet (Microsoft Office 2007) as no suitable examples were located in the available literature. For patients who had multiple general anaesthetics during one admission, data were analysed for the first surgical procedure.

### Data Collection

Patient’s vital signs data had been recorded on a number of charts: respiratory rate (admission record), temperature, heart rate and blood pressure (post-operative and routine 4-hourly charts), oxygen saturation and level of consciousness (progress report) and urine output (fluid balance chart). Data for the first eight post-operative hours were recoded into a MEWS format. Recoding was achieved by converting each recorded value (for example HR 50) for each observation time-point into a score and reporting it as normal (0) or having a low or high score of 1–3 on the review form, using the MEWS criteria (Fig. 1).

Patients’ progress notes were then searched for the nurses’ responses to triggers and other signs of disturbed physiology. Text indicative of responses to triggers included, for example, ‘Dr called’. The MEWS reporting algorithm guided interpretation of the level of urgency for calling for assistance. Recoded scores and responses were captured electronically directly onto the Excel© spreadsheet (Microsoft Office 2007) review form.

### Data Analysis

Summary statistics of the dataset for each patient were created. Bivariate comparisons were undertaken with appropriate statistical tests, determined by the distribution of the data. In cross-tabulations where one value was 0, Haldane’s estimator was used to calculate odds ratios (OR) (this circumvents 0 s in cells by adding ½ to each cell).

## Results

### Record Review

Of 2065 admissions to the six wards over the period of study, 615 (29.8%) patients had a general anaesthetic (Figure 2). Death (following cardiac arrest or unexpected death) was recorded on the hospital database for 21/615 (3.4%) patients. Of these, 4 records were unavailable, 3 were marked ‘not for resuscitation’ and 3 died outside the ward. Results relate to 11 patients who died and 44 control patients.

### Inter-rater Agreement

A 10% random sample (6/55) of anonymized reviewed records was independently coded by a nurse assessor and the first author to evaluate the quality of the clinical record review process. Agreement on the accuracy of recordings on the review form was 91.8% (56/61). Resolution of disagreement was achieved by review of selected records by both assessors and reconciliation by discussion. In a few cases legibility of symbols for graphical plotting on charts was poor especially when heart rate (indicated with a dot) intersected with the BP symbol (X).

### Patient Demographics

Demographic and clinical characteristics of the sample are presented in Tables 1–3. The mean age of patients who died (63.5 SD 10.5 years) was significantly greater than the control group (46.9 SD 16.6 years) (mean difference 16.6, t = 3.15, p, 0.003) (Table 1). Patients who died all had at least one and in some cases three or more co-morbidities, 19 control patients had no co-morbidities and no controls had 3 or more comorbidities (Table 2), otherwise the two groups were equivalent with regard to their demographic profile. Four of the 11 deaths (36.4%) were preceded by cardio-respiratory arrest (Table S2: Vital signs recordings and responses in the first 8 post-operative hours in the 11 patients who died unexpectedly on the ward).

**Table 1:** Age of the sample.

	Mean	min-max	SD	Mean difference [95% CI]	p-value	t-statistic (df)
Died (n = 11)	63.5	37–76	10.5	16.57 [6.0–27.1]	0.003	3.15 (53) Equal variances assumed
Survived (n = 44)	46.9	17–81	16.6			

Notes on table: Distributions for age are normal, therefore parametric tests are used.

**Table 2:** Clinical characteristics of the sample.

Pre-existing comorbidity^1^:	Number (%) of those who died	Number (%) of those who survived	Proportion of Sample (N = 55)		χ^2^ (df = 1)		p-value	
Myocardial infarction	1 (9.1)	1 (2.3)	2 (3.6)		Fisher’s		0.36	
Renal	2 (18.2)	1 (2.3)	3 (5.5)		Fisher’s		0.10	
Diabetes Mellitus	5 (45.5)	7 15.9)	12 (21.8)		4.50		0.03	
Carcinoma	1 (9.1)	10 (22.7)	11 (20)		1.02		0.31	
Respiratory	3 (27.3)	6 (13.6)	9 (16.4)		Fisher’s		0.36	
CVA	0	5 (11.4)	5 (9.1)		Fisher’s		0.57	
Hypertension	3 (27.3)	15 (34.1)	18 (32.7)		0.19		0.67	
1 co-morbidity	6 (54.4)	25 (56.8)	31 (56.4)	Fisher’s		1.00		
2 co-morbidities	1 (9.1)	0	1 (1.8)	Fisher’s			0.20	
3 co-morbidities	3 (27.3)	0	3 (5.5)	Fisher’s			0.01	
4+ co-morbidities	1 (9.1)	0	1 (1.8)	Fisher’s			0.20	

**Table 3:** Demographic data and type of surgery for the sample.

Characteristic	Died (n = 11)	Control/Survived (n = 44)			
	Number (%)	Number (%)	Proportion ofSample (N = 55)	χ^2^ (df = 1)	p-value
Sex: Female	4 (36.4)	29 (65.9)		3.20	0.07
Type of surgery:					
General	5 (45.5)	28 (63.6)	33 (6)		
Vascular	3 (27.3)	3 (6.8)	6 (10.9)		
Gastrointestinal	2 (18.2)	9 (20.5)	11 (20.0)		
Orthopaedic	1 (9.1)	4 (9.1)	5 (9.1)		

### Parameter Recordings within the First 8 Post-operative Hours and Nurses’ Responses

Numbers of patients with recordings of each vital sign during the 8-hour post-operative period are reported in Table 4. There was considerable variability in number of recordings for each parameter (Table S3: Number of post-operative vital signs recordings for 8 hours) therefore the median was recorded and not a mean as reported by others [38].

**Table 4:** Patients^1^ with post-operative parameter recordings by group and responses to recoded single parameter MEWS in the first 8 post-operative hours.

Parameter	Died N = 11	Survived N = 44	*x* ^2^ (df = 1)	p-value	OR (df = 1)	95% CI
	Number (%)	Number (%)				
*Respiratory rate recorded*	0	1 (2.3)	Fisher’s Exact	1.00	Not computed	
*Respiratory rate not recorded*	11 (100)	43 (97.7)				
*Respiratory rate should have triggered*	Not known	0	Not computed			
*Respiratory rate - response*	0	0				
*Heart rate recorded*	11 (100)	43 (97.7)	Fisher’s Exact	1.00	Not computed	
*Heart rate not recorded*	0	1 (2.3)				
*Heart rate should have triggered*	9 (81.8)	18 (40.9)	Fisher’s Exact	0.05	Not computed	
*Heart rate - response*	3 (3.3)	0				
*Oxygen saturation^2^ recorded*	6 (54.5)	3 (6.8)	14.65	<0.001	16.40	3.09–86.96
*Oxygen saturation not recorded*	5 (45.5)	41 (93.2)				
*Oxygen saturation should have triggered*	4 (36.4)	0	Not computed			
*Oxygen saturation - response*	2 (18.2)	0				
*Systolic BP recorded*	11 (100)	44 (100)	Not computed			
*Systolic BP not recorded*	0	0				
*Systolic BP should have triggered*	8 (72.7)	29 (65.9)	4.85	0.03	6.25	1.09–35.68
*Systolic BP - response*	4 (50.0)	4 (13.8)				
*Temperature recorded*	11 (100)	42 (95.5)	Fisher’s Exact	1.00	Not computed	
*Temperature not recorded*	0	2 (4.5)				
*Temperature should have triggered*	3 (27.3)	18 (40.9)	Fisher’s Exact	1.00	Not computed	
*Temperature - response*	0	2 (11.1)				
*Level of consciousness^3^ recorded*	4 (36.4)	30 (68.2)	3.775	0.05	0.27	0.07–1.06
*Level of consciousness not recorded*	7 (63.6)	14 (31.8)				
*Level of consciousness should have triggered*	1 (9.1)	0	Not computed			
*Level of consciousness - response*	1 (100)	0				
*Urine output recorded*	9 (81.8)	42 (95.5)	2.43	0.12	0.21	0.03–1.73
*Urine output not recorded*	2 (18.2)	2 (4.5)				
Urine output should have triggered*	6 (54.5)	14 (31.8)	Fisher’s Exact	1.00	Not computed	
*Urine output - response*	1 (16.7)	0				
*All parameters recorded*	0	0	Not computed			
*Incomplete recording of all parameters*	11	44				

Notes on table:

1. Not all patients survived for 8 hours.

2. Oxygen saturation was measured by pulse oximetry.

3. Level of consciousness denotes the patients’ state of wakefulness (‘drowsy’) usually recorded once on arrival from the operating room (taken as MEWS 0 = normal) and not the Glasgow Coma Scale assessment and should be interpreted with caution.

4. *Urine output to be interpreted with caution as estimated on fluid balance charts.

No patients in either group had recordings for all seven parameters (Table 4). All 11 patients who died had no recordings for respiratory rate; one patient in the control group (n = 44) had one recording (2.3%). All patients in both groups had recordings for systolic blood pressure. All patients who died had recordings for heart rate; in the control group all but one patient (43/44) had HR recordings (98.0%). Six patients (54.5%) in the group that died (n = 11) had recordings for oxygen saturation compared with three (6.8%) in the control group (n = 44) and this reached statistical significance (χ^2^ 14.65, df 1, p<0.001, OR 16.4, 95% CI 3.09–86.96). There were 13 recordings for oxygen saturation in the group who died; seven for the control group and this reached statistical significance (*U* = 125.5, p = <0.001), the only parameter to do so (Table S3: Number of post-operative vital signs recordings for 8 hours).

An analysis of the acuity of disturbed physiology (MEWS 1 to 3) by the number of recordings and nurses’ responses is presented in Table 5. One patient (9.1%) in the group that died (n = 11) had no abnormal parameters, as did six patients (13.6%) in the control group (n = 44), not statistically significant. Ten patients (90.9%) in the group that died (n = 11) and 38 (96.4%) patients in the control group (n = 44) had 1 to 3 abnormal parameters, not statistically significant. Six of 11 patients who died (54.5%) had 3 abnormal parameters as did five of 44 (11.4%) patients in the control group (n = 44) and this reached statistical significance (X^2^ = 10.26, df, 1, p = 0.001, OR 9.36, 95% CI 2.07–42.30).

**Table 5:** Acuity of disturbed physiology (MEWS 1 to 3)† indicating readings that triggered and should have triggered reports in the first 8 post-operative hours.

PARAMETER	Died n = 11 MEWS should have triggered No. of MEWS	Died MEWS triggered response		Survived n = 44 MEWS should have triggered No. of MEWS	Survived MEWS triggered response	
Respiratory Rate MEWS		YES (%)	NO (%)		YES (%)	NO (%)
1	0	0	0	0	0	0
2	0	0	0	0	0	0
3	0	0	0	0	0	0
Heart rate MEWS						
1	5	2 (40.0)	3 (60.0)	14	0	14 (100)
2	3	1 (33.3)	2 (66.7)	10	0	10 (100)
3	4	1 (25.0)	3 (75.0)	0	0	0
Total	12	4 (33.3)	8 (66.7)	24	0	24 (100)
Oxygen saturation MEWS						
1	2	1 (50.0)	1 (50.0)	0	0	0
2	1	0	1 (100)	0	0	0
3	2	2 (100)	0	0	0	0
Total	5	3 (60.0)	2 (40.0)	0	0	0
Systolic BP MEWS						
1	5	2 (40.0)	3 (60.0)	19	1 (5.3)	18 (94.7)
2	2	1 (50.0)	1 (50.0)	3	0	3 (100)
3	1	1 (100)	0	8	3 (37.5)	5 (62.5)
Total	8	4 (50.0)	4 (50.0)	30	4 (13.3)	26 (86.7)
Temperature MEWS						
1	0	0	0	16	1 (6.3)	15 (93.8)
2	3	0	3 (100)	3	1 (33.3)	2 (66.7)
3	0	0	0	0	0	0
Total	3	0	3 (100)	19	2 (10.5)	17 (89.5)
Conscious level MEWS						
1	1	1 (100)	0	0	0	0
2	1	1 (100)	0	0	0	0
3	0	0	0	0	0	0
Total	2	2 (100)	0	0	0	0
Urine output MEWS						
1	3	0	3 (100)	7	0	7 (100)
2	3	1 (33.3)	2 (66.7)	5	0	5 (100)
3	0	0	0	2	0	2 (100)
Total	6	1 (16.7)	5 (83.3)	14	0	14 (100)
Overall total	36	14 (38.9)	22 (61.1)	87	6 (6.9)	81 (93.1)

Notes on table:

† No distinction is made between lower and upper MEWS trigger points.

0 indicates no recordings.

10 patients (90.9%) who died (n = 11) had 1–3 parameters with abnormal MEWS: 2 (18.2%) patients had 1 abnormal parameter; 2 (18.2%) had 2 abnormal parameters; 6 (54.5%) had 3 abnormal parameters. One patient (9.1%) who died had no abnormal parameters.

In the control group (n = 44) 38 (96.4%) patients had 1–3 parameters with abnormal MEWS: 16 (36.4%) patients had 1 abnormal parameter; 17 (38.6%) had 2 abnormal parameters; 5 (11.4%) had 3 abnormal parameters. Six patients in the control group had no abnormal parameters.

There were few recordings of action taken for scores that should have been reported: there were no reports for 22/36 (61.1%) abnormal recordings for the 11 patients who died, and for 81/87 (93.1%) recordings for controls (n = 44) (Table 5). Heart rate triggered a response for three (3.3%) of nine (81.8%) patients who died (n = 11) and for 0 of 18 (40.9%) patients in the control group (n = 44) who needed assistance and this reached statistical significance (Table 4). Systolic blood pressure triggered a response for four (50.0%) of eight (72.7%) patients who died (n = 11) and for 4 (13.8%) of 29 (65.9%) patients in the control group (n = 44) who needed assistance and this reached statistical significance (Table 4). Nurses’ responses to abnormal parameters in the group of patients who died are shown in Table S2. Seven of 11 (63.3%) patients died more than 8 hours after their operation and for six of these patients, there were no recorded responses to clinical deterioration for the duration of their stay. For one of the seven patients who died 6 days after surgery, there were a number of recordings after the first 8 hours for oxygen saturation (with a MEWS of 3), a fast heart rate (n = 3 at a MEWS of 1, 2 and 3) and systolic BP (n = 1 at an upper MEWS of 1 and low MEWS of 3) but no recorded responses to these.

### Variables Associated with Mortality

Most vital sign parameters recorded on admission were not associated with post-operative death (Table 6). However, mortality was associated with: age ≥61 years (OR 14.2, 3.0–68.0), having two or more pre-existing comorbid conditions (OR 75.3, 3.7–1527.4), a high or low systolic BP on admission (OR 7.2, 1.5–34.2 three missing values in each group), a fast heart rate (OR 6.6, 1.4–30.0) and a low systolic BP (OR 8.0, 1.9–33.1) during the first 8 post-operative hours (Table 6). The association between low urine output and mortality was of borderline significance (OR 4.1, 1.0–17.3). The number of patients with recordings of respirations was too low for any inferential statistical calculation (Table 4).

**Table 6:** Factors associated with mortality between return from operating room and post-operative day 7.

Variable	Died	Survived	Association (Probability)	Odds ratio	Confidence Interval (CI)
Age category:	N = 11	N = 44	Fisher’s Exact = p<0.001	14.2†	95% 3.0–68.0†
61 years and older	9	9			
60 years and younger	2	35			
Comorbid conditions:	N = 11	N = 44	Fisher’s Exact = p<0.001	75.3†	95% CI 3.7–1527.4† ^#^
One or less	6	44			
Two or more	5	0			
Systolic BP on admission:	N = 8 (3 missing values)	N = 41 (3 missing values)	Fisher’s Exact p = 0.015	7.2†	95% CI 1.5–34.2†
High/Low systolic BP	5	7			
No High/Low systolic BP	3	34			
Heart rate 8 hourspost-operatively:	N = 11	N = 44	Fisher’s Exact p = 0.018	6.6†	95% CI 1.4–30.0
Fast heart rate (MEWS 1 to 3)	9	16			
No fast heart rate	2	28			
Systolic BP 8 hourspost-operatively:	N = 11	N = 44	Fisher’s Exact p = 0.003	8.0†	95% CI 1.9–33.1†
Low systolic BP	8	10			
No low systolic BP	3	34			
Urine output 8 hourspost-operatively:	N = 9 (2 missing values)	N = 42 (2 missing values)	Fisher’s Exact p = 0.053	4.1†	95% CI 1.0–17.3
Low urine output	6	13			
No low urine output	3	29			

Notes on table:

Unadjusted analyses.

Survivors form the reference category.

† Haldane’s estimator^222, 231.^Haldane’s estimator is used when cells have a very small or zero value. It calculates the OR as follows: ((TP+0.5)/(FN+0.5))/((FP+0.5)/(TN+0.5)): TP = true positive; FP = false positive.

# denotes that there was a 0 in one group.

## Discussion

The aim of this retrospective record review was to explore the adequacy of vital signs’ recordings in the first 8 post-operative hours and responses to clinical deterioration and to identify factors significantly associated with death on the ward between transfer from the operating room recovery suite and up to 7 days after the operation.

### Principal Findings

No patients in either group had recordings for all seven parameters listed on the MEWS. There were few post-operative recordings of vital signs. One patient in the group that died (n = 11) had no abnormal parameters, as did six patients in the control group (n = 44). Ten patients (90.9%) in the group that died and 38 (96.4%) patients in the control group had 1 to 3 abnormal parameters. Six of 11 patients who died (54.5%) had 3 abnormal vital signs as did five of 44 (11.4%) patients in the control group. There were few recordings of action taken for scores that should have been reported.

All patients who died had at least one pre-existing co-morbid condition and some had three or more which was significantly associated with mortality. Advancing age but not gender was associated with increased risk of death. An association between vital sign parameters (fast pulse rate and low systolic BP) and mortality was identified in this study.

### Limitations and Strengths of the Study in Relation to Published Studies

Uniquely, this exploration of nurses’ recordings of postoperative vital signs and responses to clinical deterioration took place in surgical wards in South Africa; together with purposive selection of the six research wards in a single research site, this limits inference of external validity [39]. These findings may not be generalisable to units where patients are monitored closely such as high dependency and intensive care.

The layout of the criterion-based review form, based on the MEWS chart, facilitated data recording, coding, extraction and analysis with speed and accuracy under field conditions. In the absence of minimum standards for recording and clinical guidelines for interpreting clinical deterioration and escalating a call for assistance, there was no standard against which to interpret the ideal number of parameter recordings or responses.

Inter-rater reliability testing of a sample of records in our study compared favourably with screening criteria for the seminal Harvard Medical Practice study [40] which revealed a sensitivity of 89% by reviewing 1% (301/30121) of reviewed records for adverse events (AEs). Our sample was larger. Review teams consist of either trained and experienced nurses and doctors [41]–[43], only doctors [40] or only nurses [44]. Our study review team comprised two nurses. In practice, nurses appear to make the initial detection of possible AEs and doctors then confirm these, and our approach reflects this [42]. The number of reviewers influences reliability. There is a higher level of agreement when a measurement is an average over several reviewers than when individual reviewers are compared and this may inflate findings [2]. Independent reviews reduce observer bias [2].

Despite the small sample size (wards and records) and the short duration of the study, we have sufficient evidence that intervention work is needed. The credence of our findings is enhanced by their similarity with those of larger studies [15], [45]–[47]. Restricting the focus of the study to mortality, the most easily defined outcome measure, limits comparisons with existing work on SAEs. Thirty patients had multiple general anaesthetics, adding to the complexity of subject selection, and leading to decisions to avoid counting the same patient twice and to analyse data for the first anaesthetic only.

The retrospective nature of this work removed volunteer bias [48], and we minimised selection bias [49]; we acknowledge the risks of bias introduced by missing data, illegibility or prior knowledge of outcomes [50]. Nevertheless, a retrospective record review meant that documentation could potentially be incomplete, for example nurses reporting abnormal vital signs verbally to senior nurses and receiving verbal instructions or nurses having telephonic discussions with the doctor that were not recorded [51].

Clinical records were compiled by clinicians prospectively, and it is unlikely that record keeping would have been influenced by unknown future outcome. However, documentation may have been influenced by nurses’ and doctors’ perceptions of the patients’ clinical condition. As in all observational studies, we cannot attribute causation. Despite these limitations, it is disconcerting that the majority of triggers (22/36) in patients who died went undocumented by any professional.

### Comparisons with other Studies

In our study mortality was associated with age (≥61 years). It is reported that SAEs, including deaths, are more common after unscheduled surgery particularly if patients are over 75 years of age, where mortality is 20% (27/135) [45], [46]. Baker et al. (2004) identified equal rates of adverse events (AEs) amongst males and females. Age-related AEs may be attributed to the complexity of care needed by older people [41].

An association between vital sign parameters (fast pulse rate and low systolic BP) and mortality was identified in this study and others [15], [32], [52]. The impact of low systolic BP is remarkably similar to another study of 79 medical emergency admissions in which the relative risk (RR 95% CI) for patients with scores of (low) 3 for systolic BP on admission compared to patients with a score of 0 was 8.6, 0.5–139 [53]. Cut points on the MEWS used in that study were similar to those of the Cape Town MEWS for systolic BP. A high incidence of recordings of disturbed physiological variables in patients in general wards has been reported [54]. Like others, we found little documented evidence of responses to early warning or even advanced signs of deterioration [55]. The proportion of unrecorded responses by nurses to signs of impending critical illness is assumed to be high.

Post-operatively, heart rate, systolic BP and temperature were plotted graphically on the existing chart, reported to portray information better than actual written values [18]. Urine output was recorded as volume in millilitres per hour as in other studies [15]. Graphic recording was reported for 90% of patients for 3739 observation sets for 189 patients in a UK retrospective record review but urine output was recorded infrequently and poorly [56]. In our study respiratory rate recordings were considerably lower than UK studies reporting recordings ranging from 73.7%, (2757/3739 observations) [56] to 44.5% (45/102 patients) [57]. Pulse oximetry measurements do not obviate the need for respiratory rate monitoring [58]. Although there were 13 pulse oximetry measurements for six patients who died in the present study, no patient who died had recordings of respiratory rate. Physiological derangements of breathing and mental status over a period of 8 hours are associated with cardiac arrest [59]. In our study significantly more patients who died had pulse oximetry measurements than those who survived.

Patients did not routinely have neurological assessments, even after general anaesthetics. Instead, recordings in patient progress notes were reported once on patients’ state of wakefulness upon return to the ward (eg. ‘drowsy’) and were recoded for interpretation in relation to the Alert/responds to voice/responds to pain/unresponsive (AVPU) classification. Reporting was poor and infrequent, as in a UK study [56]. The problems of infrequent and incomplete monitoring and recording, misinterpretation of clinical data, delays in reporting and little convincing evidence of appropriate interventions being carried out [6] were evident in this study.

Clinical decision-making involves knowledge of the biosciences, knowing the patient and learning from past experiences [60], [61]. Shearer *et al*. (2012) [62] found that the main reason nursing and medical staff did not follow rapid response system activation protocols was not inadequate cognitive interpretation of clinical deterioration but rather local sociocultural factors and intra-professional hierarchies within the clinical setting. Others [63] found that nurses did not use medical terms confidently and therefore feared looking stupid or being undermined or ridiculed and this can lead to a delay in reporting signs of deterioration. Of 110 patients who died in four Finnish hospitals, 54% had documented signs of disturbed physiology 3.8 hours before death and 11.8% of patients had no intervention [64]. Delays in calling for assistance of 1 hour have been reported for 18% patients and up to 3 hours for 8% of patients [65]. A delay in early identification of deterioration in a patient’s condition and slow transfer to ICU is associated with a 60% increase in hospitalisation costs [66].

### Meaning of the Study: Possible Mechanisms and Implications for Clinicians or Policymakers

Many SAEs occur on general wards: of 110 cardiac arrests in four Finnish hospitals, 51% (46) were on general wards [64]. To reduce SAEs at the Cape Town research setting, the policy at the time of the study made provision for routine patient admission to a High Care unit (step down from ICU) following high risk surgery and after discharge from the operating theatre recovery suite.

The standard observation chart had no criteria for identifying physiological deterioration and no criteria for activating a call for assistance. Transferring recordings to the MEWS was most useful for scoring gradations of disturbed physiology and providing guidelines for intervention in respect of each score. The limited recorded evidence of responses to deranged physiology, particularly for critically ill patients recoded as a MEWS of 3, was disturbing.

Recording too few vital signs and an inadequate number of measurements for each parameter during the first eight post-operative hours have implications for the detection of early warning signs of clinical deterioration and patient outcomes. It is recommended that a standard post-operative schedule for the frequency of recording vital signs and of the number of parameters to be recorded be adopted in public hospitals in South Africa. To improve recording and responding it is recommended that education programmes for nurses include assessment of competence in recording vital signs and summoning assistance.

There are too many confounding variables in a clinical setting to attribute mortality to poor vital signs’ monitoring alone. Nevertheless, data showing inadequate monitoring of respiratory rate, oxygen saturation, conscious level and urine output are of concern, given the associations between mortality and certain parameters [15], [32], [52]. Patients with a high or low systolic BP on admission, post-operative tachycardia and hypotension and are ≥61 years of age with two or more pre-existing comorbid conditions should be monitored most closely.

### Unanswered Questions and Future Research

We found little recorded evidence of nurses’ response to patients’ signs of deterioration. This might indicate failure to interpret vital signs’ data or be attributed to the chart not reflecting normal values for vital signs’ measurements or the absence of a reporting algorithm to guide appropriate interventions. It is recommended that the performance of existing standard observations charts used in South Africa should be tested more widely against a MEWS system for the purpose of facilitating interpretation of physiological data and responding to disturbed physiology. Future research questions are: What are the factors that contribute to nurses in a middle income developing country not reporting clinical deterioration? Will a MEWS observations chart improve recording of vital signs parameters and reporting of clinical deterioration? To ensure patient safety, the clinical community needs to know the answers to questions posed by our research, including: what is an acceptable schedule for monitoring vital signs in the immediate post-operative period following the administration of a general anaesthetic? Which vital signs parameters ought to be monitored in the immediate post-operative period?

## Conclusion

Guidelines for post-operative vital signs monitoring and reporting need to be established. The MEWS provides a useful scoring system for interpreting clinical deterioration and guiding intervention. Further research is needed to implement and explore the ability of the Cape Town MEWS chart and reporting algorithm to facilitate the recognition of signs of clinical and physiological deterioration and for summoning and securing more skilled assistance on medical and surgical wards.

## Supporting Information

Table S1 **The Strobe checklist.**
(DOC)

Table S2 **Vital signs recordings and responses in the first 8 post-operative hours for patients who died.**
(DOCX)

Table S3 **Number of post-operative vital signs recordings for 8 hours.**
(DOCX)
