# Supplementary material for: Record Review to Explore the Adequacy of Post-Operative Vital Signs Monitoring Using a Local Modified Early Warning Score (Mews) Chart to Evaluate Outcomes
Source: PLoS One. 2014 Jan 31;9(1):e87320. doi: 10.1371/journal.pone.0087320 (PMC3909075; doi:10.1371/journal.pone.0087320)
Supplement: Table S1 — The Strobe checklist. (DOC) [file pone.0087320.s001.doc]

Figure 2 STROBE Statement—Checklist of items

|  | Item  No | | Recommendation | | Application to study |  |
| --- | --- | --- | --- | --- | --- | --- |
| **Title and abstract** | 1 | (*a*) Indicate the study’s design with a commonly used term in the title or the abstract | | Found in the Title | | |
|  |  | (*b*) Provide in the abstract an informative and balanced summary of what was done and what was found | | Pg. 2 | | |
| Introduction | | | |  | | |
| Background/rationale | 2 | Explain the scientific background and rationale for the investigation being reported | | Introduction pg. 4 | | |
| Objectives | 3 | State specific objectives, including any prespecified hypotheses | | Found in the Abstract pg. 2 – no hypotheses | | |
| Methods | | | |  | | |
| Study design | 4 | Present key elements of study design early in the paper | | Methods pg. 6 | | |
| Setting | 5 | Describe the setting, locations, and relevant dates, including periods of recruitment, exposure, follow-up, and data collection | | Methods pg. 6 - 8 | | |
| Participants | 6 | (*a*) Give the eligibility criteria, and the sources and methods of selection of participants | | Methods pg. 7 and summarised in Figure 3 | | |
| Variables | 7 | Clearly define all outcomes, exposures, predictors, potential confounders, and effect modifiers. Give diagnostic criteria, if applicable | | Variables on record review form and patient demographics pg. 7-8 | | |
| Data sources/ measurement | 8* | For each variable of interest, give sources of data and details of methods of assessment (measurement). Describe comparability of assessment methods if there is more than one group | | Pg 7-8  Comparability: patients who died and those who survived | | |
| Bias | 9 | Describe any efforts to address potential sources of bias | | Pg. 9 Inter-rater reliability: A 10% random sample (6/55) of anonymized reviewed records was independently coded by a nurse assessor and the first author to evaluate the quality of the clinical record review process | | |
| Study size | 10 | Explain how the study size was arrived at | | Methods: Design pg. 6; Participants section pg. 7 | | |
| Quantitative variables | 11 | Explain how quantitative variables were handled in the analyses. If applicable, describe which groupings were chosen and why | | Methods: Data analysis pg. 8 | | |
| Statistical methods | 12 | (*a*) Describe all statistical methods, including those used to control for confounding | | Methods: data analysis pg. 8; no methods used to control for confounding as not applicable | | |
|  |  | (*b*) Describe any methods used to examine subgroups and interactions | | Not applicable | | |
|  |  | (*c*) Explain how missing data were addressed | | Table 6 | | |
|  |  | (*d*) If applicable, describe analytical methods taking account of sampling strategy | | Not applicable | | |
|  |  | (*e*) Describe any sensitivity analyses | | Not applicable | | |
| Results | | | |  | | |
| Participants | 13* | (a) Report numbers of individuals at each stage of study—eg numbers potentially eligible, examined for eligibility, confirmed eligible, included in the study, completing follow-up, and analysed | | Figure 3 for group of patients who died and the control group | | |
|  |  | (b) Give reasons for non-participation at each stage | | Not applicable | | |
|  |  | (c) Consider use of a flow diagram | | Figure 3 | | |
| Descriptive data | 14* | (a) Give characteristics of study participants (eg demographic, clinical, social) and information on exposures and potential confounders | | Tables 1, 2, 3 | | |
|  |  | (b) Indicate number of participants with missing data for each variable of interest | | Table 6 | | |
| Outcome data | 15* | Report numbers of outcome events or summary measures | | Tables 4, 5, 6 | | |
| Main results | 16 | (*a*) Give unadjusted estimates and, if applicable, confounder-adjusted estimates and their precision (eg, 95% confidence interval). Make clear which confounders were adjusted for and why they were included | | Table 6: Unadjusted analyses | | |
|  |  | (*b*) Report category boundaries when continuous variables were categorized | | Not applicable | | |
|  |  | (*c*) If relevant, consider translating estimates of relative risk into absolute risk for a meaningful time period | | Not computed | | |
| Other analyses | 17 | Report other analyses done—eg analyses of subgroups and interactions, and sensitivity analyses | | Only data relevant to study objectives are reported | | |
| Discussion | | | |  | | |
| Key results | 18 | Summarise key results with reference to study objectives | | Pg. 8 - 11 | | |
| Limitations | 19 | Discuss limitations of the study, taking into account sources of potential bias or imprecision. Discuss both direction and magnitude of any potential bias | | Pg. 11 | | |
| Interpretation | 20 | Give a cautious overall interpretation of results considering objectives, limitations, multiplicity of analyses, results from similar studies, and other relevant evidence | | Pg. 11-14 | | |
| Generalisability | 21 | Discuss the generalisability (external validity) of the study results | | Pg. 11 | | |
| Other information | | | |  | | |
| Funding | 22 | Give the source of funding and the role of the funders for the present study and, if applicable, for the original study on which the present article is based | | Pg. 15 | | |

*Give information separately for exposed and unexposed groups.
