# Supplementary material for: Record Review to Explore the Adequacy of Post-Operative Vital Signs Monitoring Using a Local Modified Early Warning Score (Mews) Chart to Evaluate Outcomes
Source: PLoS One. 2014 Jan 31;9(1):e87320. doi: 10.1371/journal.pone.0087320 (PMC3909075; doi:10.1371/journal.pone.0087320)
Supplement: Table S2 — Vital signs recordings and responses in the first 8 post-operative hours for patients who died. (DOCX) [file pone.0087320.s002.docx]

Table S2: Vital sign recordings and responses in the first 8 postoperative hours in the 11 patients who died unexpectedly on the ward

| Died (n=11) | Age | Postoperative period of death | Number of abnormal readings in first 8 hours post-operatively | Number of times assistance was summoned |
| --- | --- | --- | --- | --- |
| 1 | 72 | Peri-arrest: 15 hours 15 minutes; death: 19 hours 40 minutes – unsuccessful CPR | RR x 0 recordings, HR x 2 MEWS=3 upper + 1 low SBP MEWS=1 low, Temp x 1 MEWS=2 low, UO MEWS=1 | Once: HR + SBP |
| 2 | 62 | 52 hours | RR x 0 recordings, SBP x 1 low, Temp x 2 low | Once for SBP |
| 3 | 67 | 11 hours, Apnoea 1 hour prior to death – no reporting; Intern attended – unsuccessful CPR | RR x 0 recordings, SATS x 1, HR x 1 MEWS 1 low, SBP x 1 MEWS 1 low | 0 |
| 4 | 55 | 2 days | RR x 0 recordings, HR x 1 MEWS 3 upper, SBP x 1 MEWS 2, Temp x 1 MEWS 2 upper | 0 |
| 5 | 70 | 42 hours – unsuccessful CPR | RR x 0 recordings, HR x 1 MEWS upper, UO x 1 MEWS 2 low | 0 |
| 6 | 63 | 6 days – unsuccessful CPR | RR x 0 recordings, SATS x 1 MEWS 3*, HR x 3 MEWS 1 upper, 2 upper*, 3 upper*, SBP x 1 MEWS 1 upper*, 3 low* | 0 |
| 7 | 37 | 3 hours 20 minutes – declared dead by doctor (no CPR) | RR x 0 recordings, SATS x 1, HR x 3 MEWS 2 upper, SBP x 1 MEWS 1 low, AVPU MEWS 1 – 2 restless tried to climb out of bed | Once for AVPU coinciding with faecaloid emesis |
| 8 | 64 | 3 hours – unsuccessful CPR | RR x 0 recordings, SATS x 2 MEWS 1, 3, HR x 3 MEWS 2 high, 3 upper x 2, SBP x 1 MEWS 1 low | Once for SATS |
| 9 | 70 | 4 days – unsuccessful CPR | RR x 0 recordings, SATS x 1 MEWS 3 | 0 |
| 10 | 76 | 11 hours – unsuccessful CPR | RR x 0 recordings, HR x 1 MEWS 2 upper, SBP x 1 MEWS 3 low | Once for HR, Once for SBP |
| 11 | 62 | 7 days – unsuccessful CPR | RR x 0 recordings, SATS x 1 MEWS 3, HR x 1 MEWS 1 upper, SBP x 1 MEWS 2 low, UO x 1 MEWS 2 low | Once for SATS, Once for HR |

Note on table: * Responses after the first 8 postoperative hours
