# Supplementary material for: Record Review to Explore the Adequacy of Post-Operative Vital Signs Monitoring Using a Local Modified Early Warning Score (Mews) Chart to Evaluate Outcomes
Source: PLoS One. 2014 Jan 31;9(1):e87320. doi: 10.1371/journal.pone.0087320 (PMC3909075; doi:10.1371/journal.pone.0087320)
Supplement: Table S3 — Number of post-operative vital signs recordings for 8 hours. (DOCX) [file pone.0087320.s003.docx]

Table S3: Number of postoperative vital signs recordings (for 8 hours)

|  |  | | | | Mann-Whitney U test | | | | | | | |
| --- | --- | --- | --- | --- | --- | --- | --- | --- | --- | --- | --- | --- |
| Parameter | Number of recordings | | | | Mean rank | | | Sum of Ranks | U statistic | | Z-value | p-value |
| *RESPIRATORY RATE* | Total number^1^ | Median | Min-max | |  |  |  |  |  |  |  |  |
| Died (n=11) | 0 | 0 | 0-0 | | 27.50 | | | 302.50 | 236.50 | | -.50 | 0.62 |
| Survived (n=44) | 1 | 0 | 0-1 | | 28.13 | | 1237.50 | | |  |  |  |
| *HEART RATE* | | | | | | | | | | | | |
| Died (n=11) | 80 | 7 | | 2-13 | | 33.32 | | 366.50 | 183.50 | | -1.24 | 0.22 |
| Survived (n=44) | 272 | 6 | | 0-14 | | 26.67 | | 1173.50 |  |  |  |  |
| *OXYGEN SATURATION* | | | | | | | | | | | | |
| Died (n=11) | 13 | 1 | | 0-3 | | 38.59 | | 424.50 | 125.50 | | -3.81 | <0.001 |
| Survived (n=44) | 7 | 0 | | 0-5 | | 25.35 | | 1115.50 |  |  |  |  |
| *SYSTOLIC BLOOD PRESSURE* | | | | | | | | | | | | |
| Died (n=11) | 92 | 9 | | 3-15 | | 33.86 | | 372.50 | 177.50 | | -1.36 | 0.17 |
| Survived (n=44) | 305 | 7 | | 2-14 | | 26.53 | | 1167.50 |  |  |  |  |
| *TEMPERATURE* | | | | | | | | | | | | |
| Died (n=11) | 19 | 2 | | 1-3 | | 24.27 | | 267.00 | 201.00 | | -.93 | 0.35 |
| Survived (n=44) | 94 | 2 | | 0-5 | | 28.93 | | 1273.00 |  |  |  |  |
| *CONSCIOUS LEVEL^2^* | | | | | | | | | | | | |
| Died (n=11) | 5 | 0 | | 0-2 | | 22.36 | | 246.00 | 180.00 | | -1.53 | 0.13 |
| Survived (n=44) | 30 | 1 | | 0-1 | | 29.41 | | 1294.00 |  |  |  |  |
| *URINE OUTPUT* | | | | | | | | | | | | |
| Died (n=11) | 25 | 2 | | 0-8 | | 32.59 | | 358.50 | 191.50 | | -1.13 | 0.26 |
| Survived (n=44) | 72 | 1 | | 0-7 | | 26.85 | | 1181.50 |  |  |  |  |

Note on table: Total number of observations = all observation time-points.

1. State of wakefulness recorded in patient progress notes and not on the observations chart.
